# Venous thromboembolism following colectomy for diverticular disease: an English population-based cohort study

**DOI:** 10.1007/s00423-023-02920-6

**Published:** 2023-05-22

**Authors:** Anjali K D S Yapa, David J Humes, Colin J Crooks, Christopher A Lewis-Lloyd

**Affiliations:** 1 Gastrointestinal Surgery, National Institute for Health Research (NIHR) Nottingham Biomedical Research Centre (BRC), Nottingham University Hospitals NHS Trust and the University of Nottingham, School of Medicine, Queen’s Medical Centre, Nottingham, UK; 2 Gastrointestinal & Liver Theme, National Institute for Health Research (NIHR) Nottingham Biomedical Research Centre (BRC), Nottingham University Hospitals NHS Trust and the University of Nottingham, School of Medicine, Queen’s Medical Centre, Nottingham, UK

**Keywords:** Venous thromboembolism, Diverticular disease, Colectomy, Emergency

## Abstract

**Aim:**

This study reports venous thromboembolism (VTE) rates following colectomy for diverticular disease to explore the magnitude of postoperative VTE risk in this population and identify high risk subgroups of interest.

**Method:**

English national cohort study of colectomy patients between 2000 and 2019 using linked primary (Clinical Practice Research Datalink) and secondary (Hospital Episode Statistics) care data. Stratified by admission type, absolute incidence rates (IR) per 1000 person-years and adjusted incidence rate ratios (aIRR) were calculated for 30- and 90-day post-colectomy VTE.

**Results:**

Of 24,394 patients who underwent colectomy for diverticular disease, over half (57.39%) were emergency procedures with the highest VTE rate seen in patients ≥70-years-old (IR 142.27 per 1000 person-years, 95%CI 118.32–171.08) at 30 days post colectomy. Emergency resections (IR 135.18 per 1000 person-years, 95%CI 115.72–157.91) had double the risk (aIRR 2.07, 95%CI 1.47–2.90) of developing a VTE at 30 days following colectomy compared to elective resections (IR 51.14 per 1000 person-years, 95%CI 38.30–68.27). Minimally invasive surgery (MIS) was shown to be associated with a 64% reduction in VTE risk (aIRR 0.36 95%CI 0.20–0.65) compared to open colectomies at 30 days post-op. At 90 days following emergency resections, VTE risks remained raised compared to elective colectomies.

**Conclusion:**

Following emergency colectomy for diverticular disease, the VTE risk is approximately double compared to elective resections at 30 days while MIS was found to be associated with a reduced risk of VTE. This suggests advancements in postoperative VTE prevention in diverticular disease patients should focus on those undergoing emergency colectomies.

**Supplementary Information:**

The online version contains supplementary material available at 10.1007/s00423-023-02920-6.

## Introduction

Diverticular disease is one of the main indications for colectomy in both the emergency and elective setting [1, 2]. This condition and its complications are becoming more common with increasing hospital admissions being reported in the United States of America (US) and United Kingdom (UK) [3–5]. Following this rise in complications of diverticular disease, the rates of elective colectomies for diverticulitis have subsequently increased by 29% over a 7-year period [6, 7].

Current literature reports patients with diverticular disease and those undergoing colorectal surgery, especially as an emergency procedure are at a greater risk of postoperative venous thromboembolism (VTE) [8–11]. Therefore, VTE rates are expected to be high in this group [12]. However, recent changes in perioperative care such as the increased use of minimally invasive surgery (MIS) and enhanced recovery after surgery (ERAS) guidelines may have affected VTE risk. Current guidelines on VTE prevention following colectomy, used globally in standard practice, target patient groups who are known to be at high risk for VTE such as those with cancer and to a lesser extent Inflammatory Bowel Disease (IBD) [13]. However, there is a paucity of guidance for patients being managed surgically for benign abdominal disease, in particular diverticulitis, with no specific guidance on extended thromboprophylaxis in this group [14, 15]. With cases of surgically managed diverticular disease rising worldwide, this gap in the guidelines could leave patients at significant risk.

Existing studies explore VTE rates following colectomy [16–19]; however, reports on VTE following surgical management of diverticular disease are scarce. This novel study uses longer follow up timeframes as well as explores the effect of MIS techniques on colectomy for diverticular disease to supplement the current literature [9, 12, 20–24]. The aim of this study was to explore the rates of VTE in patients undergoing surgery for diverticular disease and determine at risk subgroups to inform current guidelines.

## Methods

The study was approved by the Independent Scientific Advisory Committee approval board (Protocol 19_180RA3).

### Data sources

Three validated and linked healthcare databases were used for this study: The Clinical Practice Research Datalink (CPRD) GOLD, CPRD Aurum and Hospital Episode Statistics Admitted Patient Care (HES APC) databases [25–27]. The CPRD databases house primary care data including diagnoses, referrals and prescriptions for 60 million patients collected from General Practitioner practices across the UK [28]. The HES APC database records all admissions to National Health Service (NHS) and independent sector hospitals. Patient admission data regarding discharge diagnosis and procedures are coded using the International Statistical Classification of Diseases and Related Health Problems 10th Revision (ICD-10) codes and the Office of Population, Censuses and Surveys Classification of Surgical Operations and Procedures version 4 (OPCS-4) codes respectively [29, 30].

### Cohort

OPCS-4 codes from HES data linked to CPRD GOLD and Aurum practices were used to identify patients that underwent colectomy between the years 2000 and 2019. Patient data had to meet the research standard and have coinciding data collection time periods for both primary and secondary databases to be included within the validated cohort. Patients with diverticular disease undergoing colectomy were identified using the HES ICD-10 codes (see Supplementary 1). The exclusion criteria used encompassed completely endoscopic operations, those confined to the anal canal, patients less than 18 years old and those with previous personal history of VTE. Patients identified as having a VTE event prior to colectomy were excluded due to their inherently increased risk of VTE [31] as outlined in the patient flow chart (see supplementary 1). Person-time and postoperative follow-up started the day after the date of operation. All follow-up lasted until earliest date of VTE event, death, and change to a non-participating general practice or 90 days from the date of operation.

### Exposures

The main exposures of interest were admission type, sex, operative technique, and co-morbidity. From HES data, admission type was classified as either elective or emergency. For emergency admissions, those with diverticular disease with perforation and/or abscess were included using ICD-10 codes (Supplementary 1) and were classed as perforated or not perforated. Operative technique included colectomies that were carried out open or started using a minimally invasive approach. Minimally invasive techniques were defined as laparoscopic or robotic surgery using OPCS-4 codes (Y50.8, Y57.1, and Y75.2) and (Y75.3) respectively. Defined from HES data, sex was either male or female and length of stay was the time in days from date of operation to discharge. Patients who had stomas were defined using OPCS-4 codes and patient age in years at colectomy was categorized into <60, 60–69, and ≥70. CPRD and HES data reported patient co-morbidity using the Charlson score with categories 0, 1, and ≥2 co-morbidities [30].

### Outcomes

A diagnosis of a post-colectomy VTE event was the primary outcome. Post-colectomy VTE was defined as a VTE event occurring after colectomy from either medical or ICD-10 codes in the linked CPRD and HES datasets. To be considered valid VTE events, evidence of treatment in an anticoagulation clinic (for example a medical code) within a period of 15 days before and 90 days after VTE diagnosis, a prescription for anticoagulant medication or a date of death within 30 days of the event was required. Furthermore, VTE diagnosis also included instances where VTE was identified as an underlying cause of death and only the first confirmed VTE event was included within the analysis. This definition using primary care data has been formerly validated [19] and used in previous studies [27].

### Statistical analysis

Cohort demographics were presented as proportions and stratified by admission type. Absolute incidence rates (IR) of VTE were calculated per 1000 person-years with 95% confidence intervals (95%CI). Post-colectomy VTE rates and adjusted incidence rate ratios (aIRR) for linear trend at 30 and 90 days post colectomy, adjusted for age, sex, co-morbidity, admission type, and operative technique were calculated using Poisson regression and stratified *a priori* where possible by admission type. For emergency admissions, the calculated VTE rates and aIRR were also adjusted for perforation and abscess. The cumulative incidence of VTE was calculated up to 90 days using Kaplan-Meier curves.

All data management and analyses were performed using Stata SE® version 17.0 (StataCorp LLC, College Station, Texas, USA).

## Results

### Cohort demographics

Of 24,394 patients who underwent colectomy for diverticular disease, over half (57.39%) were performed as emergency procedures. These patients were older (≥70 years old 45.22%) and had ≥2 significant co-morbidities (53.08%). There was a similar number of male and female patients undergoing colectomy as an emergency (43.68% vs 56.32%) or elective (44.14% vs 55.86%) procedure. In the emergency setting, 78.61% of patients undergoing colectomy had a stoma while 25.03% of patients had a stoma in the elective group. Overall, the open operative technique was used more frequently in both emergency (91.64%) and elective (64.08%) settings. (See Table 1) However, over the study period there was a notable rise in the rates of MIS. In the elective population, MIS rates increased from 0.87% in the year 2000 to 68.47% (*p*<0.0001) in 2019. Similarly, the rate of emergency MIS in 2000 was 0.22% and by 2019 was 18.87% (*p*<0.0001). The median length of stay in the elective and emergency groups were 8 (Interquartile range (IQR) 6–12) days and 13 (IQR 8–22) days respectively.

**Table 1:** Demographics of colectomy cohort by admission type

	Total (No. = 24,394 )			
	Emergency		Elective	
	No.	(%)	No.	(%)
	13,999	(57.39)	10,395	(42.61)
Age (Years)				
< 60	4,693	(33.52)	4,100	(39.44)
60 − 69	2,975	(21.25)	2,917	(28.06)
≥ 70	6,331	(45.22)	3,378	(32.50)
Sex				
Male	6,115	(43.68)	4,588	(44.14)
Female	7,884	(56.32)	5,807	(55.86)
Charlson Score				
0	5,393	(38.52)	4,274	(41.12)
1	1,176	(8.40)	1,011	(9.73)
≥ 2	7,430	(53.08)	5,110	(49.16)
Operative Technique				
Open	12,828	(91.64)	6,661	(64.08)
Started MIS	1,171	(8.36)	3,734	(35.92)
Perforation				
No perforation	4,270	(30.50)	-	-
Perforation	9,729	(69.50)	-	-
Stoma formation				
Yes	11,004	(78.61)	2,602	(25.03)
No	2,995	(21.39)	7,793	(74.97)

### VTE rates at 30 days

The VTE rate following colectomy at 30 days was 98.76 per 1000 person years (95%CI 86.12–113.25).

Patients who underwent MIS had a 64% reduced risk of VTE (aIRR 0.36 95% CI 0.20–0.65) compared to those who underwent open colectomies at 30 days (Table 2).

**Table 2:** Rates of venous thromboembolism at 30 days post colectomy

	Event no.	Person-years	Rate per 1000 person-years	95% CI	IRR (uni)	95% CI	IRR (multi)	95% CI
Elective	46	0.90	51.14	38.30–68.27	1.00	(ref)	1.00	(ref)
Emergency	159	1.18	135.18	115.72–157.91	2.64	1.90–3.67	2.07	1.47–2.90
Age								
<60	47	0.77	61.07	45.88–81.27	1.00	(ref)	1.00	(ref)
60–69	45	0.51	87.91	65.64–117.74	1.44	0.96–2.17	1.35	0.89–2.05
≥70	113	0.79	142.27	118.32–171.08	2.33	1.66–3.27	1.88	1.30–2.72
Sex								
M	77	0.92	83.81	67.03–104.78	1.00	(ref)	1.00	(ref)
F	128	1.16	110.63	93.03–131.56	1.32	1.00–1.75	1.11	0.83–1.49
Charlson score								
0	68	0.84	81.12	63.96–102.88	1.00	(ref)	1.00	(ref)
1	17	0.19	91.88	57.12–147.80	1.13	0.67–1.93	1.02	0.59–1.73
≥2	120	1.05	114.02	95.34–136.36	1.41	1.04–1.89	1.14	0.84–1.56
Operative technique								
Open	193	1.65	116.96	101.57–134.68	1.00	(ref)	1.00	(ref)
Started MIS	12	0.43	28.19	16.01–49.64	0.24	0.13–0.43	0.36	0.20–0.65

**Fig. 1:**
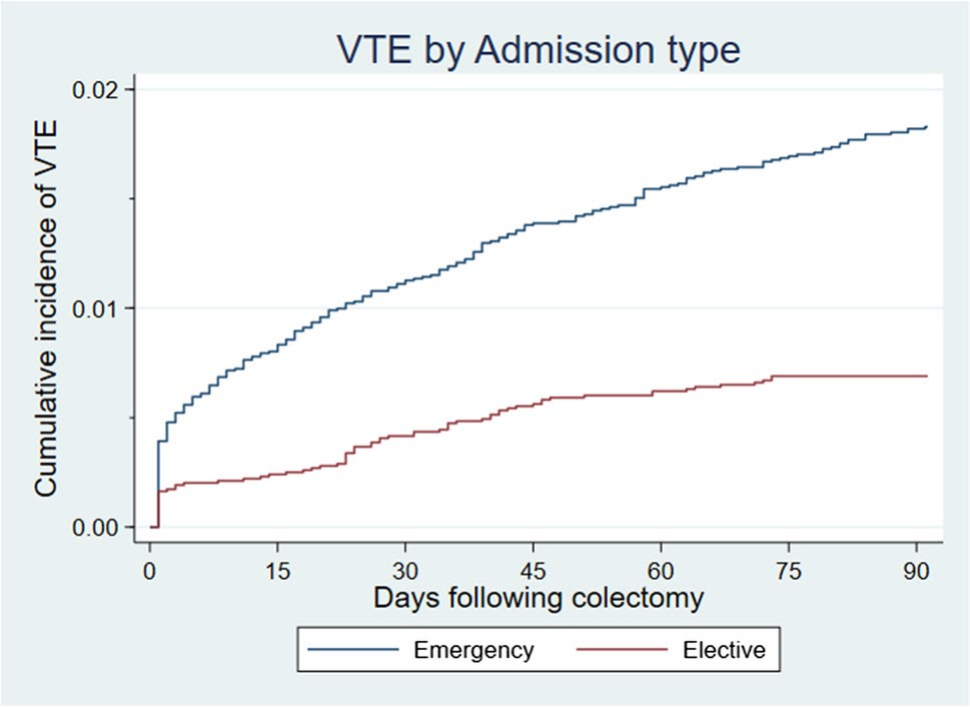
Cumulative Incidence curve for post colectomy venous thromboembolism rates by time post admission, stratified by admission type. At 30 days, the cumulative incidence was 0.42% and 1.11% for elective and emergency colectomy respectively (*p*<0.001). At 90 days, the cumulative incidence was 0.69% and 1.82% for elective and emergency colectomy respectively (*p*<0.001)

Following elective colectomy, the overall rate of VTE at 30 days was 51.14 per 1000 person-years (95%CI 38.30–68.27) and 135.18 per 1000 person-years (95%CI 115.72–157.91) following emergency colectomy (Table 2). Patients undergoing emergency colectomy had a twofold increased risk (aIRR 2.07, 95%CI 1.47–2.90) of developing VTE 30 days post-surgery when compared to those in the elective group. These differences remained significant after adjusting for age, gender, co-morbidity, and operative technique. The cumulative incidence of VTE at 30 days were 1.11% in the emergency setting and 0.42% (*p*<0.001) following elective colectomy (see Fig 1).

#### VTE rates at 90 days

The overall VTE rate following colectomy at 90 days was 54.72 per 1000 person years (95%CI 48.96–61.15). All VTE rates at 90 days post-op were stratified by admission type as shown below, as there were sufficient VTE events to allow for this.

#### Emergency colectomy

**Fig. 2:**
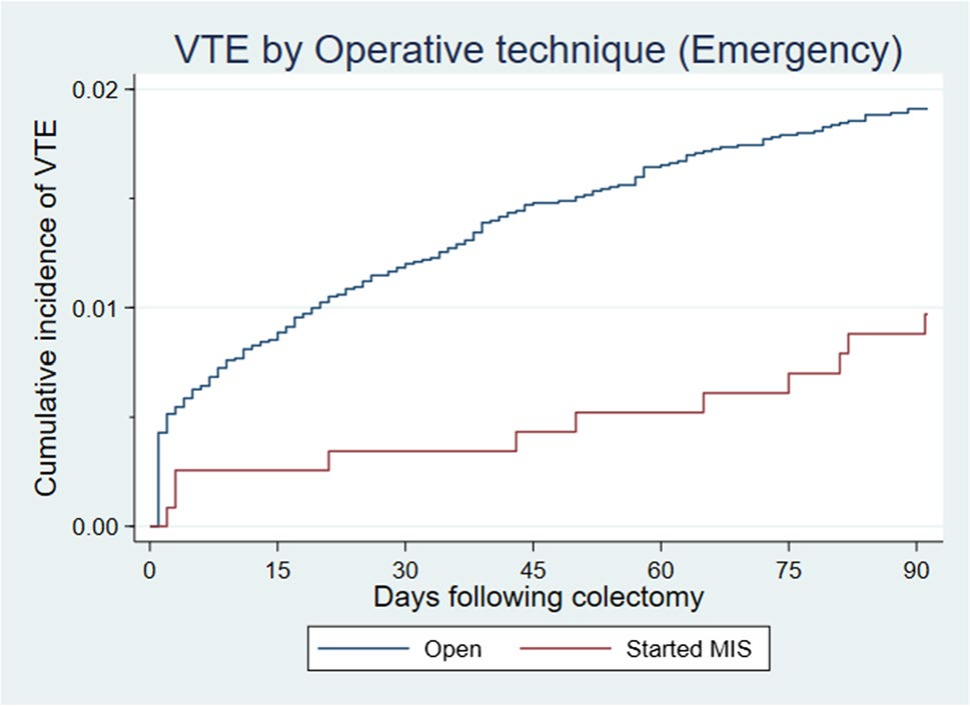
Cumulative Incidence curve for post-surgery venous thromboembolism rates following emergency colectomy by time post admission, stratified by operative technique: At 30 days, the cumulative incidence was 0.34% and 1.18% (*p*=0.008) for emergency MIS and open surgery respectively. At 90 days, the cumulative incidence was 0.88% and 1.91% (*p*=0.015) for emergency MIS and open surgery respectively. MIS, minimally invasive surgery

In our model for the emergency setting, age was the only factor that had a significant effect on VTE rates at 90 days. Table 3 shows that emergency patients ≥70 years old had VTE rates almost 3 times greater (aIRR 2.97, 95% CI 2.05–4.29) than those <70 years old. An almost 50% rise in the crude rate of VTE was seen in female patients (90.7 per 1000 person-years, 95% CI 77.48–106.16) compared to males (59.46 per 1000 person-years, 95% CI 48.01–73.64). The crude rate of VTE was lower post emergency MIS at 90 days (38.1 per 1000 person-years (95% CI 21.11–68.82)) than it was following an open approach (80.5 per 1000 person-years (95% CI 70.68–91.63), translating to a non-significant but almost 50% reduction in VTE risk following MIS colectomy (aIRR 0.57 95%CI 0.31–1.04). Although the crude VTE rates were higher in patients with perforation and/or abscess when compared to those without perforation, this reduced after adjusting for the other factors in the model and was not significant (aIRR 1.27 95%CI 0.95–1.68). Figure 2 shows a cumulative incidence of 0.88% of VTE events following MIS and 1.91% (*p*=0.015) following open surgery at 90 days post-op. Overall, 1.82% of emergency colectomy patients developed post-op VTE at 90 days.

**Table 3:** Rates of venous thromboembolism at 90 days post-surgery in patients undergoing emergency colectomy

Emergency								
	Event no.	Person-years	Rate per 1000 person-years	95% CI	IRR (uni)	95% CI	IRR (multi)	95%CI
Age								
<60	42	1.15	36.48	26.96–49.36	1.00	(ref)	1.00	(ref)
60–69	47	0.69	68.06	51.14–90.59	1.87	1.23–2.83	1.76	1.15–2.69
≥ 70	150	1.28	117.21	99.88–137.56	3.21	2.28–4.52	2.97	2.05–4.29
Sex								
M	84	1.41	59.46	48.01–73.64	1.00	(ref)	1.00	(ref)
F	155	1.71	90.70	77.48–106.16	1.53	1.17–1.99	1.17	0.89–1.54
Charlson score								
0	78	1.28	60.93	48.80–76.07	1.00	(ref)	1.00	(ref)
1	19	0.26	73.68	47.00–115.51	1.21	0.73–2.00	0.96	0.58–1.60
≥2	142	1.58	89.67	76.07–105.70	1.47	1.12–1.94	1.08	0.81–1.45
Operative technique								
Open	228	2.83	80.48	70.68–91.63	1.00	(ref)	1.00	(ref)
Started MIS	11	0.29	38.11	21.11–68.82	0.47	0.26–0.87	0.57	0.31–1.04
Perforation								
No perforation	68	0.97	70.41	55.52–89.30	1.00	(ref)	1.00	(ref)
Perforation	171	2.16	79.32	68.28–92.14	1.13	0.85–1.49	1.27	0.95–1.68

#### Elective colectomy

**Fig. 3:**
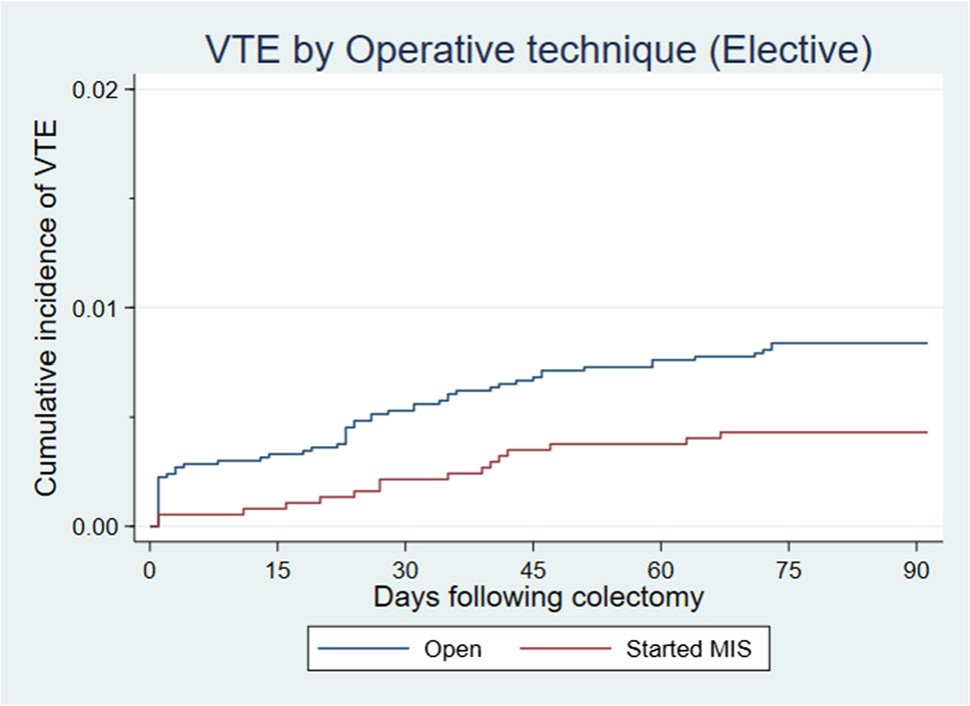
Cumulative Incidence curve for post-surgery venous thromboembolism rates following elective colectomy by time post admission, stratified by operative technique**.** At 30 days, the cumulative incidence was 0.21% and 0.53% (*p*=0.013) for elective MIS and open surgery respectively. At 90 days, the cumulative incidence was 0.43% and 0.84% (*p*=0.016) for elective MIS and open surgery respectively. MIS, minimally invasive surgery

Age, gender, and comorbidity did not have a significant effect on the rate of VTE episodes 90 days post elective colectomy. Table 4 shows a minimally invasive approach resulted in 47% reduction in VTE risk (aIRR 0.53, 95% CI 0.30–0.93) compared to an open approach at 90 days. Additionally, a cumulative incidence of 0.43% and 0.84% (p=0.016) is shown for VTE events 90 days post MIS and open colectomy respectively in Fig. 3. Overall, 0.69% of elective colectomy patients developed post-op VTE at 90 days.

**Table 4:** Rates of venous thromboembolism at 90 days post-surgery in patients undergoing elective colectomy

Elective								
	Event no.	Person-years	Rate per 1000 person -years	95% CI	IRR (uni)	95% CI	IRR (multi)	95% CI
Age								
<60	21	1.02	20.59	13.43–31.59	1.00	(ref)	1.00	(ref)
60-69	22	0.72	30.42	20.03–46.20	1.48	0.81–2.69	1.33	0.72–2.45
≥ 70	29	0.82	35.40	24.60–50.94	1.72	0.98–3.01	1.45	0.80–2.63
Sex								
M	25	1.13	22.12	14.95–32.74	1.00	(ref)	1.00	(ref)
F	47	1.43	32.83	24.66–43.69	1.48	0.91–2.41	1.32	0.80–2.17
Charlson score								
0	26	1.06	24.51	16.69–35.99	1.00	(ref)	1.00	(ref)
1	10	0.25	40.35	21.71–74.99	1.65	0.79–3.41	1.55	0.75–3.23
≥2	36	1.25	28.72	20.72–39.82	1.17	0.71–1.94	1.05	0.63–1.76
Operative technique								
Open	56	1.64	34.23	26.34–44.48	1.00	(ref)	1.00	(ref)
Started MIS	16	0.93	17.28	10.59–28.20	0.50	0.29–0.88	0.53	0.30–0.93

## Discussion

### Summary

In this cohort of patients undergoing colectomy for diverticular disease, the overall rate of VTE was found to be higher following emergency colectomy. Furthermore, the rates for both emergency and elective admissions continue to rise with time post-surgery. The proportion of emergency colectomy patients that develop VTE following surgery climbs from 1.11 to 1.82% at 30 and 90 days respectively. In those ≥70 years, the VTE rates were 3 times greater (aIRR 2.97, 95% CI 2.05–4.29) than patients <70 years at 90 days post emergency colectomy. This is in comparison to a rise from 0.42 to 0.69% of patients that develop VTE following elective colectomy at 30 and 90 days respectively where age was not a significant factor. Conversely, when looking at operative technique, MIS is associated with lower VTE rates. In the 90 days post elective colectomy, the rate of VTEs was 17.3 per 1000 person-years (95% CI 10.59–28.20) and 34.2 per 1000 person-years (95% CI 26.34–44.48) following MIS and open surgery respectively. Although the results were not significant, a similar reduction is seen when comparing VTE rates post emergency colectomy at 90 days for MIS and open surgery. These findings demonstrate that patients, particularly the elderly, undergoing open emergency colectomy for diverticular disease have a prolonged VTE risk and would therefore potentially benefit from VTE prevention strategies, such as extended VTE prophylaxis.

## Strengths and limitations

Being a large population study, this study has the power to allow observed VTE rates to be stratified by admission type, operative technique and known VTE risk factors. Additionally, by using an unselected population-based cohort, its outcomes will also be generalizable to other populations with universally accessible modern healthcare systems. To avoid any surveillance bias [32], a validated definition of VTE was used to capture both inpatient and outpatient VTE events from both primary and secondary care. This ensured the cohort was not comprised of solely hospitalized patients [27].

A limitation of this study was the unavailability of patient level hospital prescribing information on NHS Direct, and therefore it was not possible to assess the effect of VTE prophylaxis regimen directly. However, data from a recent national audit reports risk assessments and therefore appropriateness of VTE prophylaxis for 95–96% of NHS acute admissions were carried out [33, 34]. Consequently, patients in this cohort would have had VTE prophylaxis prescribed in keeping with national trends thereby, not affecting the interpretation of our results. As with any population-based database, there are always concerns surrounding the accuracy of recorded data. However, the databases utilized within this study have been extensively used, previously validated and contain built-in metrics to assure data quality and accuracy, including the use of a validated definition of a VTE outcome [19, 27, 35, 36].

### Emergency colectomy

Overall, the rate of VTE is doubled at 30 days following emergency colectomies when compared to those done in an elective setting. Furthermore, patients over 70 years old had a threefold increased risk of VTE at 90 days following emergency colorectal surgery. This coincides with multiple studies that have found emergency surgery to be a risk factor for post-operative VTEs and yield almost double the rates when compared to their elective counterparts [10, 17, 19, 37, 38]. While these studies investigate the occurrence of post-colectomy VTEs, they do not explore those related to diverticular disease in particular or the effect of MIS. Some studies [17] had shorter investigation periods and only included in-patient VTE events. More recently, a large quality improvement project by Poulos et al. explored likely subgroups at increased risk of VTE following diverticular resection [12]. Over an 11-year period, they too found emergency surgery and age > 65 to be significant predictors of VTE events.

### Elective colectomy

There are no prior studies specifically addressing the VTE risk following elective surgery in patients with diverticular disease. Operative technique is a significantly associated with VTE events at 90 days following elective colorectal resection [39]. The crude VTE rates following MIS and open surgery were 17.3 and 34.2 per 1000 person years respectively which translates to a halved risk of VTE post MIS. Other studies report a similar reduction in VTE rates following minimally invasive colectomy [40–43]. Shapiro et al. [40] demonstrated rates of 1.2% and 2.9% following laparoscopic and open approaches however this population included colorectal cancer and IBD and was not specific to diverticular disease. Meanwhile, over a 5 year period Mohadamyeghaneh et al. [42] found open colorectal surgery was associated with a 33% increased risk of deep venous thromboembolism and 73% increased risk of pulmonary embolism when compared to a laparoscopic approach. However, this study focused solely on VTE rates post colectomy for perforated diverticulitis.

## Clinical relevance

As the incidence of diverticular disease increases [45, 46] emergent colectomies are becoming more frequent [6, 47]. Emergency colorectal surgery and diverticular disease are known independent risk factors for VTE [9, 48]. Despite this rising prevalence, post-colectomy VTE rates for diverticular disease in particular have not been previously explored. Our cohort of patients with surgically managed disease had significant VTE rates at 30 (98.76 per 1000 person years, 95% CI 86.12–113.25) and 90 (54.72 per 1000 person years 95% CI 48.96–61.15) days following colectomy. Emergent colectomy had a subsequently greater VTE risk at 30 days while those over 70 years old had VTE rates 3 times greater than other age groups at 90 days after colectomy. These clear high-risk groups identified within patients with surgically managed diverticular disease may also be potential targets for future interventional studies in mitigating this increased risk. Minimally invasive alternatives as well as the implementation of ERAS have been shown to reduce the risk of developing post-colectomy VTE and have better surgical outcomes [42–44, 49–54].

## Conclusion

VTE rates are elevated following emergency colorectal surgery for diverticular disease in comparison to elective colectomy. These observed VTE rates were elevated at both 30 and 90 days post-op. As they are at an increased risk for VTE, patients undergoing emergency colectomy for diverticular disease may represent a high risk group for future interventional studies for example on the use of extended VTE prophylaxis.

### Supplementary information

ESM 1 26.7 KB
