# Supplementary material for: Venous thromboembolism following colectomy for diverticular disease: an English population-based cohort study
Source: Langenbecks Arch Surg. 2023 May 22;408(1):203. doi: 10.1007/s00423-023-02920-6 (PMC10203000; doi:10.1007/s00423-023-02920-6)
Supplement: Supplementary file 1 — ESM 1 26.7 KB [file 423_2023_2920_MOESM1_ESM.docx]

Venous Thromboembolism Following Colectomy for Diverticular Disease: An English Population Based Cohort Study (Supplementary 1)

Running head: Rates of VTE after colectomy for diverticular disease

Dr. Anjali K D S Yapa BmedSci (Hons) BMBS, Mr. David J Humes^1^ PhD FRCS, Dr. Colin J Crooks BmedSci (Hons) BMBS^2^, Dr. Christopher A Lewis-Lloyd^1^ BmedSci (Hons) BMBS (Hons)

^1^Gastrointestinal Surgery, National Institute for Health Research (NIHR) Nottingham Biomedical Research Centre (BRC), Nottingham University Hospitals NHS Trust and the University of Nottingham, School of Medicine, Queen’s Medical Centre, Nottingham, United Kingdom.

^2^Gastrointestinal & Liver Theme, National Institute for Health Research (NIHR) Nottingham Biomedical Research Centre (BRC), Nottingham University Hospitals NHS Trust and the University of Nottingham, School of Medicine, Queen’s Medical Centre, Nottingham, United Kingdom.

Corresponding author: Dr Anjali K D S Yapa

Postal address and affiliation: Department of Gastrointestinal Surgery, National Institute for Health Research (NIHR) Nottingham Biomedical Research Centre (BRC), Nottingham University Hospitals NHS Trust and the University of Nottingham, School of Medicine Queen’s Medical Centre, Derby Road, Nottingham, NG7 2UH, United Kingdom

Emails: [Anjali.dias96@gmail.com](mailto:Anjali.dias96@gmail.com), Telephone: +44 1158231153

Supplementary 1

ICD-10 codes used to identify bowel perforation with diverticular disease.

Perforated: K57.01, K57.41, K63.1, K57.21, K57.0, K57.2, K57.00, K57.20, K57.81, K57.80, K57.40, K57.41, K57.4, K57.8

Non-perforated: K57.13, K57.33, K57.12, K57.32, K57.52, K57.53, K57.92, and K57.93

OPCS codes used to identify colectomy:

Colectomy: H041, H042, H043, H048, H049, H051, H052, H053, H058, H059, H061, H062, H063, H064, H068, H069, H071, H072, H073, H074, H078, H079, H081, H082, H083, H084, H085, H088, H089, H091, H092, H093, H094, H095, H098, H099, H101, H102, H103, H104, H105, H108, H109, H111, H112, H113, H114, H115, H118, H119, H291, H292, H293, H294, H298, H299, H331, H332, H333, H334, H335, H336, H337, H338, H339

Patient flow chart with inclusion & exclusion criteria: below

Patient data meets research standard and data collection times coincide for both HES and CPRD GOLD databases

(n=18,366)

Patient data meets research standard and data collection times coincide for both HES and CPRD Aurum databases (n=165,111)

Combined HES linked CPRD GOLD and Aurum patient data up to research standard

(n=183,477)

Patients undergoing colectomy for diverticular disease identified using ICD-10 codes between 2000 and 2019

(n=24,394)

Patients undergoing elective colectomy (n=10,395)

Patients undergoing emergency colectomy (n=13,999)

Linked HES and CPRD GOLD cohort of patients undergoing colectomy between 2000-2019

(n=49,388)

Linked HES and CPRD Aurum cohort of patients undergoing colectomy between 2000-2019

(n=246,240)

Excluded:

-did not meet research standards

-patients undergoing completely endoscopic operations

-operations confined to anal canal

-age <18 years

-previous personal history of VTE events
